# A Novel Approach to Differentiating Erosive and Reticular Lichen Planus Based on the Percentage of Dental Surfaces With Metal Restorations

**DOI:** 10.7759/cureus.44782

**Published:** 2023-09-06

**Authors:** Vasileios Zisis, Nikolaos N Giannakopoulos, Marc Schmitter, Athanasios Poulopoulos, Dimitrios Andreadis

**Affiliations:** 1 Prosthodontics, Julius-Maximilians-Universität Würzburg, Würzburg, DEU; 2 Oral Medicine/Pathology, Aristotle University of Thessaloniki, Thessaloniki, GRC

**Keywords:** oral metal restoration, oral lichenoid lesion, oral lichenoid reaction, exposure to metal index, oral lichen planus

## Abstract

Introduction

Oral lichen planus (OLP) and oral lichenoid reaction (OLR) constitute clinical entities with strong but unclear etiologic relation to dental materials. The aim of this study was to evaluate a correlation between the clinical form of OLP/OLR and the number of dental metal restorations in the oral cavity thus utilizing an exposure to metal (EM) index.

Material and methods

The study type is experimental, and the study design is characterized as semiquantitative research that belongs to the branch of experimental research. Twenty-nine patients were chosen based on clinical (either reticular or erosive clinical forms) and histologic findings suggestive of OLP/OLR. The files of patients were retrieved from the archives of the Department of Oral Medicine/Pathology, School of Dentistry, Aristotle University of Thessaloniki, Greece, during the period 2009-2019. The medical history of the patients did not include any disorder or medication associated with lichenoid lesions and the measurements took place concurrently with the establishment of the diagnosis, thus no treatment for the lichen planus had been administered prior to the measurements. Quantitative measurement of the percentage of dental surfaces restored through metal restorations and correlation with the clinical and histologic findings of OLP/OLR was evaluated. The EM index was evaluated on a scale of 1-3, which corresponds to the percentage of dental surfaces restored through metal restorations. The statistical analysis was performed with the Pearson chi-square test and the significance level was set at *p*≤0.05.

Results

The EM index was measured by dividing each tooth into five surfaces (occlusal, mesial, distal, buccal, lingual), subsequently multiplying the number of available teeth with the number 5 to calculate the total number of surfaces, and then counting the number of surfaces with metal restorations - both fillings and crowns (in case of metal-ceramic crowns, the respective dental surface is taken into account only in case of macroscopically exposed metal), dividing the number of surfaces with metal restorations with the total number of surfaces and multiply by 100 so that the results take the form of percentages (%) and finally classifying the percentages into three groups: 1: 0% metal restorations, 2: 1-25% metal restorations, 3: >26% metal restorations). The percentage in female patients ranged from 0% to 100%, whereas it ranged from 0% to 60% in male patients. According to the clinical form of the lichenoid lesion, the percentage ranged from 0% to 60% in reticular lichen planus cases and from 0% to 100% in erosive lichen planus cases. There was no statistical difference between lichen planus cases, in total, and in normal oral epithelium. However, the levels of EM were marginally similar between the reticular lichen planus and the erosive lichen planus (Fisher’s exact test, *p = 0.056*). Therefore, it may be the case that the EM index is higher in erosive lichenoid lesions.

Conclusion

In our study, the EM index was higher in female patients and in erosive lichenoid lesions. These findings should be tested and supported by larger samples of patients since the aforementioned Fisher’s Exact Test,* p = 0.056 *could fall below the threshold of 0.05 if more patients were included. This is the first attempt to establish a novel approach to differentiating erosive and reticular lichen planus based on the percentage of dental surfaces with metal restorations.

## Introduction

Lichen planus (LP) is a relatively common, chronic, autoimmune, mucocutaneous disease that often affects the oral mucosa but can also affect the skin, scalp, genitalia, and nails [1-2]. The cutaneous lesions expand like lichens (algae and fungi) on rocks [3]. Of course, despite its name, lichen planus is not a fungal condition but is an immunologically mediated disorder. Oral lichen planus (OLP) is an ongoing (chronic) inflammatory condition that affects mucous membranes inside the oral cavity. OLP, which has been characterized as an oral, potentially malignant disorder by the WHO [4], more commonly affects middle-aged patients than children [5-6] and especially affects females during the sixth and seventh decade of their lives [7-8] rather than males, usually by a 3:2 ratio [9]. OLP can clinically manifest itself through seven subtypes: popular, reticular, atrophic, erosive, plaque-like, bullous, and pigmentosus [10].

The oral lichenoid reaction (OLR) is clinically similar to the OLP, and its pathogenesis is immunologically mediated, OLP-like, and involves the lymphocytic infiltration of T-cells [11]. The OLP histology is characteristic and OLRs may share some histopathologic features, being difficult or impossible to separate from OLP. The main histopathologic features of lichen planus are the dense subepithelial lymphocytic and histiocytic infiltration, the destruction of the basal cell layer, the degeneration apoptosis of the basal keratinocytes of the epithelium (hydropic degeneration), and the intraepithelial presence of lymphocytes [12]. Subjacent to the epithelium, an intense, bandlike infiltrate of predominantly T lymphocytes is observed. The apoptotic keratinocytes’ remnants form colloid bodies (otherwise named Civatte bodies) that appear as homogenous eosinophilic bodies [13]. It is interesting to note that exposure to materials leads to an immunologic reaction that may mimic or cross-lead to OLP/OLR.

Exposure to nickel leads to activation of the CD4+ T-cells after the nickel ions are presented to the MHC II complex by the APCs [14]. These ions react with the nitrogen and oxygen in amino acid chains of the MHC II complex producing protein-metal complexes [15]. The effect of nickel on CD8+ T-cells remains unknown [14]. Chronic exposure to palladium may lead to a generalized activation of the TH1 and TH2 CD4+ T-cells and to increased levels of IL-1α, IL-4, IL-6, IL-10, IL-12, GM-CSF, and INF-γ [16]. Furthermore, exposure to palladium facilitates the expression of NKG2D by CD8⁺ T cells, which is a costimulatory molecule involved in the production of IFN-γ [17]. Exposure to copper is associated with decreased numbers of CD4+ T-cells and increased numbers of CD8+ T-cells [18]. CD4+ T-cells are activated because of exposure to CoCr (cobalt/chromium) alloys [19] while the expression of CD8+ T-cells was upregulated and increased levels of IL-6 and TNFα were noticed [20]. Beryllium binds to HLA-DP molecules (binding spot: glutamic acid at position 69 of the β-chain) and creates a characteristic complex, thus forming the beryllium-specific, Th1 CD4+ T-cells, which are further associated with granuloma formation in the lung [21]. These pathways shape the immunological background, which in turn frames their undistinguishable microscopic appearance [22].

The aim of this study is to establish a new, innovative approach to the establishment of the diagnosis and prognosis of OLP/OLR. The group of the normal epithelium functions as the control group to illustrate the differences between OLP/OLR and the normal epithelium.

## Materials and methods

The study type is experimental, and the study design is characterized as semiquantitative research that belongs to the branch of experimental research. In this study, 24 patients with confirmed OLP/OLR based on clinical and histologic findings were included along with five patients, diagnosed without any lichenoid lesions (patients had reactive lesions-fibromas), forming the group of normal oral epithelium. The files of the patients were retrieved from the archives of the Department of Oral Medicine/Pathology, School of Dentistry, Aristotle University of Thessaloniki, Greece, during the period 2009-2019. The study was conducted in accordance with the guidelines of the Research and Ethics Committee of Aristotle University, School of Dentistry, and the Helsinki II declaration. The present study was approved by the Ethics Committee of the School of Dentistry, Aristotle University of Thessaloniki, Greece (8/03.07.2019). The study included 22 female patients and seven male patients whose ages ranged from 21 to 81 years old. The inclusion criteria were the balanced distribution of the locations of the lesions involved (tongue, cheek, lips, floor of the mouth, gingiva, corner of the mouth) and specifically for the oral lichen planus and normal oral epithelium patients, the presence of metal restorations in the oral cavity for more than 10 years (according to the patient report). The exclusion criterion was the presence of metal restorations in the oral cavity for less than 10 years (according to the patient report). Subsequently, they were examined to measure the percentage of dental surfaces restored through metal restorations and correlate it with the histopathological diagnosis of erosive lichen planus due to dental restorative materials and with reticular lichen planus. The percentage was categorized into three groups corresponding to a scale of 1-3 of the EM (exposure to metal) index as shown in Table 1.

**Table 1:** Classification of the percentages of surfaces with metal restorations into the EM (exposure to metal) index scale of 1-3

Percentage of surfaces with metal restorations	EM (exposure to metal) index
0%	1
1-25%	2
>26%	3

The EM index is calculated by dividing each tooth into five surfaces (occlusal, mesial, distal, buccal, lingual), subsequently multiplying the number of available teeth with the number 5 to calculate the total number of surfaces, and then counting the number of surfaces with metal restorations both fillings and crowns (in case of metal-ceramic crowns, the respective dental surface is taken into account only in case of macroscopically exposed metal), dividing the number of surfaces with metal restorations with the total number of surfaces and multiply by 100 so that the results take the form of percentages (%) and finally classifying the percentages into three groups: 1: 0% metal restorations, 2: 1-25% metal restorations, 3: >26% metal restorations. The restrictions of the EM index include the fact that the exact time of the placement of the metal restorations in the oral cavity was unknown and the study depended on the patient report, the former dental history regarding previous treatments with metal restorations could not be taken into account, as well as the exact composition of the metal restorations, and the changes of saliva pH, which affects the erosion of the metal restorations through time. Also, the adverse effects of medication taken by the patient, regarding the saliva production and its rate, were not possible to be counted. In addition, the vicinity of surfaces with metal restorative materials and oral mucosa was not taken into account. Statistical analysis was performed using SPSS software 2017 (SPSS Inc., Chicago, IL) with the Pearson chi-square test and Fisher’s exact test depending on the sample size. The significance level was set at 0.05 (p=0.05). Statistical analysis was performed to compare the exposure to metal index between four pairs: lichen planus to normal oral epithelium, erosive lichen planus to reticular lichen planus, erosive lichen planus to normal oral epithelium, and reticular lichen planus to normal oral epithelium.

## Results

The percentage of dental surfaces restored through metal restorations in female patients ranged from 0% to 100%, whereas in male patients, it ranged from 0% to 60%. According to the clinical form of lichenoid lesions, the percentage ranged from 0% to 60% in the reticular lichen planus cases and from 0% to 100% in the erosive lichen planus cases. In the normal oral epithelium group, the percentage ranged from 0-40%. Regarding the EM index, two samples of the reticular lichen planus group were scored as 1, seven samples of the reticular lichen planus group were scored as 2 and one sample of the reticular lichen planus group was scored as 3. Two samples of the erosive lichen planus group were scored as 1, four samples of the erosive lichen planus group were scored as 2, and eight samples of the erosive lichen planus group were scored as 3. Three samples of the normal oral epithelium group were scored as 1, one sample of the normal oral epithelium group was scored as 2, and one sample of the normal oral epithelium group was scored as 3 (Table 2).

**Table 2:** Summary of the distribution of EM index levels EM: exposure to metal

	OLP		Normal Oral epithelium	Total
	Reticular OLP	Erosive OLP		
EM index 1	2	2	3	7
EM index 2	7	4	1	12
EM index 3	1	8	1	10
Total	10	14	5	29

**Figure 1:**
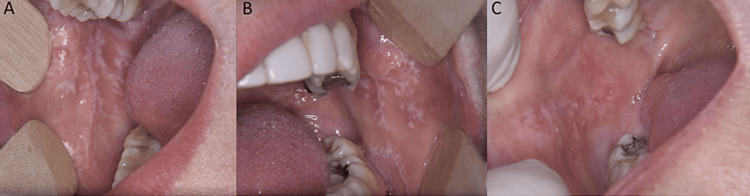
Typical clinical appearance of lichen planus in the oral cavity A: Reticular lichen planus; B: Reticular lichen planus in close proximity to metal restorations; C: Erosive lichen planus in close proximity to metal restorations

The patients included in the study, as well as their demographical details, are summarized in Table 3. Figure 1 depicts relevant clinical pictures of the patients included in the study.

**Table 3:** Summary of the demographical details of the patients included in the study

Patients	Category	Location	Gender	Age
1	Lichen planus/reticular	Tongue	Male	57
2	Lichen planus/reticular	Tongue	Male	77
3	Lichen planus/reticular	Tongue	Female	21
4	Lichen planus/reticular	Tongue	Female	50
5	Lichen planus/reticular	Tongue	Female	57
6	Lichen planus/reticular	Cheek	Female	72
7	Lichen planus/reticular	Tongue	Female	38
8	Lichen planus/reticular	Cheek	Male	73
9	Lichen planus/reticular	Cheek	Female	49
10	Lichen planus/reticular	Cheek	Female	42
11	Lichen planus/erosive	Interdental papilla	Female	77
12	Lichen planus/erosive	Cheek	Female	77
13	Lichen planus/erosive	Tongue	Female	59
14	Lichen planus/erosive	Cheek	Female	54
15	Lichen planus/erosive	Palate	Female	55
16	Lichen planus/erosive	Cheek	Female	49
17	Lichen planus/erosive	Cheek	Female	72
18	Lichen planus/erosive	Cheek	Female	76
19	Lichen planus/erosive	Cheek	Female	58
20	Lichen planus/erosive	Cheek	Female	64
21	Lichen planus/erosive	Cheek	Male	56
22	Lichen planus/erosive	Cheek	Male	56
23	Lichen planus/erosive	Gingiva	Female	26
24	Lichen planus/erosive	Cheek	Female	37
25	Normal	Tongue	Female	49
26	Normal	Cheek	Male	81
27	Normal	Cheek	Female	59
28	Normal	Tongue	Male	69
29	Normal	Tongue	Female	72

**Figure 2:**
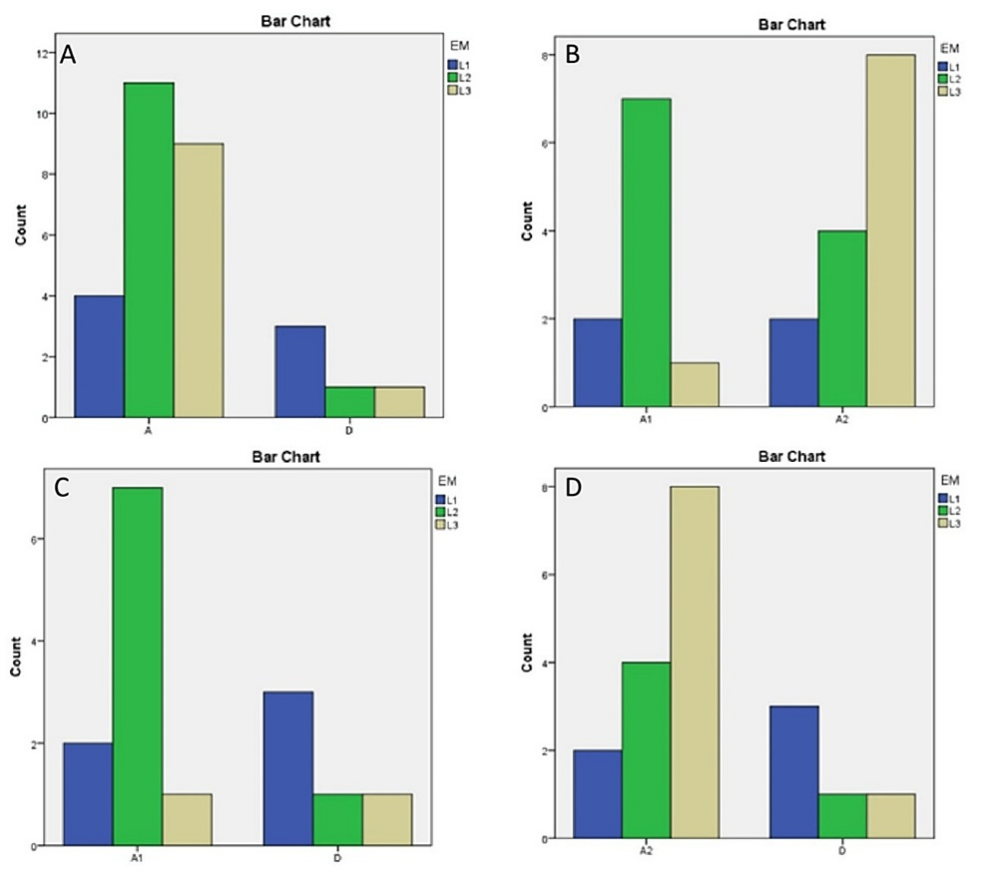
Summary of the bar charts of the statistical analysis L1-L3 on the y-axis represent the number of patients per the EM index level. A, D, A1, and A2 on the x-axis represent OLP, normal oral epithelium, reticular OLP, and erosive OLP, respectively. 1A describes the comparison between OLP and normal oral epithelium. 1B describes the comparison between reticular OLP and erosive OLP. 1C describes the comparison between reticular OLP and normal oral epithelium. 1D describes the comparison between erosive OLP and normal oral epithelium. EM: exposure to metal

The chi-squared tests that were performed did not show any statistically significant difference between OLP and normal oral epithelium regarding the levels of the EM index (Fisher’s exact test, p = 0.168), between reticular OLP and erosive OLP regarding the levels of the EM index (Fisher’s exact test, p = 0.056), between reticular OLP and normal oral epithelium regarding the levels of the EM index (Fisher’s exact test, p = 0.231), and between erosive OLP and normal oral epithelium regarding the levels of the EM index (Fisher’s exact test, p = 0.180) (Table 4, Figure 2).

**Table 4:** Summary of the statistical results of the study Special notice to the marginally similar results between reticular and erosive OLP.

	OLP - Normal	Reticular OLP - Normal	Erosive OLP - Normal	Reticular OLP - Erosive OLP
p-values	p = 0.168	p = 0.231	0.180	p = 0.056

## Discussion

The prevalence of OLP depending on the population ranges from 0.02% to 1.2% [2,23]. The global pooled prevalence amounts to 1.01%, with the highest prevalence reported from Europe (1.43%) [24]. Ninety-three percent (93%) of OLRs were significantly ameliorated when amalgam was removed and replaced [25]. Mercury-containing amalgam provokes an immune response, leading to apoptosis of basal keratinocytes and to increased risk of malignant transformation [25]. Dental cast alloys (containing nickel, gold, palladium, cobalt, and copper) and dental amalgam (containing mostly mercury, silver, tin, and copper with some variations, including zinc, indium, palladium, and platinum) may trigger OLR and gingival inflammation [26-30]. Porcelain, composite, and glass ionomer cement may also trigger OLR [27-28,30-32]. The molecules of HEMA, Bis-GMA, and methacrylate resins, all known contents of composite resins, cause OLR, despite the inhibition of free molecules by light curing [33]. While on routine surveillance or after being attracted by chemokines, antigen-specific CD8+cytotoxic T-cells enter the oral epithelium and trigger apoptosis of basal keratinocytes, provoking OLP. The initial contact between T-cells and the keratinocyte antigen takes place either when the CD8+T-cell accidentally encounters the keratinocyte antigen while it moves through the epithelium (chance encounter hypothesis) [34-35] or it migrates on purpose to the site where the keratinocyte antigen is present, attracted by chemokines secreted by the keratinocyte expressing the antigen (directed migration hypothesis) [12].

The aim and research hypothesis of this study was to establish a new approach to the establishment of diagnosis and prognosis of OLP/OLR. So far, the differential diagnosis between OLP and OLR has proven to be dubious. At a histological level, the two entities are indistinguishable. The location of the lesions, the proximity to dental restorations, and the symmetrical, intraoral distribution of the lesions were taken into account for the purpose of the differential diagnosis. However, these criteria acted as a logical leap since they cannot be characterized as pathognomonic and only functioned on a probabilistic level. Since, by definition, the differentiation between OLP and OLR remains unclear, the novel approach under investigation emphasizes the general exposure to metal restorations and, in particular, the percentage of dental surfaces with metal restorations in the oral cavity. Higher levels of cytotoxicity are noticed in the presence of metal restorations. Furthermore, higher levels of cytotoxicity are noticed in the presence of erosive lichenoid lesions. A logical assumption to be tested statistically may be whether the erosive lichenoid lesions are positively correlated to the presence of metal restorations. For this purpose, the classification of the erosive lichenoid lesions into one group is based solely on their clinical features and the presence of metal restorations in the oral cavity of the patients involved to avoid confusion regarding their histological features and their histological distinction, and this group may hence be termed as the erosive lichen planus group. The proposed novel approach determines the clinical diagnosis and prognosis based on the percentage of dental surfaces with metal restorations and the chronic exposure of the oral epithelium to metal alloys.

The two main drawbacks of the EM index to be reported were that the exact composition of the metal restorations was unknown and that only the present situation was taken into account when calculating the percentage of dental surfaces with metal restorations. The inclusion criterion that the metal restorations were present in the oral cavity for at least 10 years was met (according to the patient's report), but nevertheless, the exact duration of the chronic exposure to metals was unknown. Another innovative element is that we compared not only oral lichen planus with normal oral epithelium but also its respective subgroups, reticular OLP and erosive OLP. Such a study has not been reported in the literature so far. The evidence, originating from our study, showed that the levels of EM were higher in the erosive lichen planus group compared to the reticular lichen planus group.

Limitations

The limitations of the study included the lack of an independent group of OLP consisting of patients without any metal restorations, the disproportionate participation of female patients, the fact that the exact time of the placement of the metal restorations in the oral cavity is unknown, the study depended on the patient's report, the former dental history regarding previous treatments with metal restorations was not taken into account, the exact composition of the metal restorations was unknown, the effect of pH, which affects the erosion of metal restorations, in the oral cavity was not taken into account, the quantity of saliva and the rate of saliva production was not taken into account, and finally, the adverse effects of medication taken by the patient, regarding the saliva production, was not taken into account. Finally, the vicinity of surfaces with metal restorative materials and oral mucosa was not taken into account.

## Conclusions

The exposure to metal index showed promising results in distinguishing different subtypes of oral lichen planus enhancing the certainty of the diagnosis combined with the histological examination. Solid suggestions for future research would be the application of this index to larger numbers of patients, its modification to include more information from the medical history, and its application in oral leukoplakia cases to investigate whether the immunological response and the inflammation instigated by the metal restorations is a prerequisite for the development of epithelial dysplasia.
